# Supplementary material for: Nutrients, Diet, and Other Factors in Prenatal Life and Bone Health in Young Adults: A Systematic Review of Longitudinal Studies
Source: Nutrients. 2020 Sep 19;12(9):2866. doi: 10.3390/nu12092866 (PMC7551661; doi:10.3390/nu12092866)
Supplement: Supplementary file 1 [file nutrients-12-02866-s001.zip › Table S2 .docx]

**Table S2: Search strategy for “Nutrients, Diet and Other Factors in Prenatal Life and Bone Health in Young Adults: A Systematic Review of Longitudinal tudies”**

**The search strategy used to search MEDLINE**

Filters activated: Clinical Study, Clinical Trial, Systematic Reviews, Review, Randomized Controlled Trial, Observational Study, Multicenter Study, Meta-Analysis, Humans, English, Danish, Field: Title/Abstract.

| **Fetal life** | **AND** | **Exposure** | **AND** | **Outcome** |
| --- | --- | --- | --- | --- |
| “Fetal life”  Fetus (MeSH terms)  Fetus  Foetus  Fetal  Foetale  Pregnant  Pregnancy  Pregnancy (MeSH terms)  Offspring  Programming  “Fetal Programming”  Fetal development (MeSH terms)  “Fetal development”  Embryo and fetal development (MeSH terms)  Foetal  “Maternal-fetal Environment”  Maternal-Fetal Exchange (MeSH terms)  Periconception  Periconceptions  Periconceptional  Periconceptionally  Maternal  Prenatal  Prenatally  Prenatal exposure delayed effects (MeSH terms) |  | Diet (MeSH terms)  Diet  Diets  Foods  Food (MeSH terms)  Food  Food and beverages (MesH terms)  Nutrient  Nutrients (MeSH terms)  Nutrients  Supplement  Supplements  Supplemental  Dietary supplements (MeSH terms)  “Dietary supplements”  BMI  Body mass index (MeSH terms)  “Body mass index”  “Waist circumference”  Waist circumference (MeSH terms)  Exercise (MeSH terms)  Exercise  “Physical activity”  Smoking (MeSH terms)  Smoking  Alcohols (MeSH terms)  Alcohols  Alcohol  Drug  Drugs  ”Drug abuse”  ”Drug abuser”  Medicine  Asthma (MeSH terms)  Asthma  Asthmatic  Allergy and immunology (MeSH terms)  Preeclampsia  Pre-eclampsia (MeSH terms)  Pre-eclampsia  “Gestational diabetes”  Diabetes, gestational (MeSH terms)  “Pregnancy in diabetics” (MeSH terms)  Pregnancy complications (MeSH terms)  “Pregnancy complications”  “Pregnancy-related complications”  “Gestational weight gain” Gestational weight gain (MeSH terms)  “Maternal health”  Maternal health (MeSH terms)  Maternal behavior (MeSH terms)  Anthropometry (MeSH terms)  Anthropometry  Maternal exposure (MeSH terms)  “Maternal exposure” |  | Bone  “Bone and Bones” (MeSH terms)  Fracture  Fractures  “Bone fractures”  “Bone fracture”  “Fractures risk”  “Fracture risk”  Fractures, Bone (MeSH terms)  “Bone mineral density”  Bone density (MeSH terms)  “Bone density”  BMD  Osteopenia  “Trabecular bone score”  TBS  “Bone mass”  “Bone mineral apparent density”  “Bone mineral content”  BMC  “Bone health”  “Peak bone mass”  PBM  “Bone loss”  “Reduced bone mass” |

**The search strategy used to search EMBASE**

Clinical Study, Clinical Trial, Systematic Reviews, Review, Randomized Controlled Trial, Observational Study, Multicenter Study, Meta-Analysis, Humans, English, Danish, Field: Title/Abstract.

| **Fetal life** | **AND** | **Exposure** | **AND** | **Outcome** |
| --- | --- | --- | --- | --- |
| “Fetal life”  Fetus  Foetus  Fetal  Foetale  Pregnant  Pregnancy  Offspring  Programming  “Fetal Programming”  “Fetal development”  Foetal  “Maternal-fetal Environment”  Periconception  Periconceptions  Periconceptional  Periconceptionally  Maternal  Prenatal  Prenatally  Prenatal exposure (Emtree terms)  Fetus (Emtree terms)  Pregnancy (Emtree terms)  Fetal development (Emtree terms) |  | Diet  “dietary supplements”  Foods Diets  Nutrient  Nutrients  Supplement  Supplemental  Supplements  BMI  “Body mass index”  “Waist circumference”  Anthropometry  Exercise  “Physical activity”  Smoking  Alcohol  Alcohols  Drug  Drugs  “Drug abuse”  “Drug abuser”  “Maternal exposure”  “Maternal health”  “Gestational weight gain”  “Pregnancy-related complications”  Dietary supplement (Emtree terms)  Diet (Emtree terms)  Food (Emtree terms)  Beverages (Emtree terms)  Nutrient (Emtree terms)  Body mass (Emtree terms)  Anthropometry (Emtree terms)  Waist circumference (Emtree terms)  Exercise (Emtree terms)  Maternal smoking (Emtree terms)  Smoking (Emtree terms)  Alcohol (Emtree terms)  Alcohol abuse (Emtree terms)  Alcohol consumption (Emtree terms)  Drug abuse (Emtree terms)  Maternal exposure (Emtree terms)  Maternal behavior (Emtree terms)  Maternal welfare (Emtree terms)  Gestational weight gain (Emtree terms)  Pregnancy complication (Emtree terms)  Pregnancy diabetes mellitus (Emtree terms)  Preeclampsia (Emtree terms)  Asthma (Emtree terms)  Allergy (Emtree terms) |  | Bone  “Bone mass”  Fracture  Fractures  “Bone fractures”  “Bone fracture”  “Fractures risk”  “Fracture risk”  “Bone mineral density”  “Bone density”  BMD  “Trabecular bone score”  TBS  “Bone mineral apparent density”  “Bone mineral content”  BMC  “Bone health”  “Peak bone mass”  PBM  “Bone loss”  “reduces bone mass”  Bone (Emtree terms)  Bone mass (Emtree terms)  Fracture (Emtree terms)  Bone density (Emtree terms) |

**The search strategy used to search Cochrane Library.**

Filters activated: Reviews, English, Danish

| **Fetal life** | **AND** | **Exposure** | **AND** | **Outcome** |
| --- | --- | --- | --- | --- |
| “Fetal life”  Fetus (MeSH terms)  Fetus  Foetus  Fetal  Foetale  Pregnant  Pregnancy  Pregnancy (MeSH terms)  Offspring  Programming  “Fetal Programming”  Fetal development (MeSH terms)  “Fetal development”  Embryo and fetal development (MeSH terms)  Foetal  “Maternal-fetal Environment”  Maternal-Fetal Exchange (MeSH terms)  Periconception  Periconceptions  Periconceptional  Periconceptionally  Maternal  Prenatal  Prenatally  Prenatal exposure delayed effects (MeSH terms) |  | Diet (MeSH terms)  Diet  Diets  Foods  Food (MeSH terms)  Food  Food and beverages (MeSH terms)  Nutrient  Nutrients (MeSH terms)  Nutrients  Supplement  Supplements  Supplemental  Dietary supplements (MeSH terms)  Dietary supplementations (MeSH terms)  Dietary supplements (MeSH terms)  Dietary supplements”  BMI  Body mass index (MeSH terms)  “Body mass index”  “Waist circumference”  Waist circumference (MeSH terms)  Exercise (MeSH terms)  Exercise  “Physical activity”  Smoking (MeSH terms)  Smoking  Alcohols (MeSH terms)  Alcohols  Alcohol  Drug  Drugs  ”Drug abuse”  ”Drug abuser”  Medicine  Asthma (MeSH terms)  Asthma  Asthmatic  Allergy and immunology (MeSH terms)  Preeclampsia  Pre-eclampsia (MeSH terms)  Pre-eclampsia  “Gestational diabetes”  Diabetes, gestational (MeSH terms)  “Pregnancy in diabetics” (MeSH terms)  Pregnancy complications (MeSH terms)  “Pregnancy complications”  “Pregnancy-related complications”  “Gestational weight gain” Gestational weight gain (MeSH terms)  “Maternal health”  Maternal health (MeSH terms)  Maternal behavior (MeSH terms)  Anthropometry (MeSH terms)  Anthropometry  Maternal exposure (MeSH terms)  “Maternal exposure” |  | Bone  “Bone and Bones” (MeSH terms)  Fracture  Fractures  “Bone fractures”  “Bone fracture”  “Fractures risk”  “Fracture risk”  Fractures, Bone (MeSH terms)  “Bone mineral density”  Bone density (MeSH term)  “Bone density”  BMD  Osteopenia  “Trabecular bone score”  TBS  “Bone mass”  “Bone mineral apparent density”  “Bone mineral content”  BMC  “Bone health”  “Peak bone mass”  PBM  “Bone loss”  “Reduced bone mass” |
