# Supplementary material for: Nutrients, Diet, and Other Factors in Prenatal Life and Bone Health in Young Adults: A Systematic Review of Longitudinal Studies
Source: Nutrients. 2020 Sep 19;12(9):2866. doi: 10.3390/nu12092866 (PMC7551661; doi:10.3390/nu12092866)
Supplement: Supplementary file 1 [file nutrients-12-02866-s001.zip › Table S3.docx]

# Table S3: List of excluded studies after full text screening and reasons for their exclusion in “Nutrients, Diet, and Other Factors in Prenatal Life and Bone Health in Young Adults: A Systematic Review of Longitudinal Studies” [1-230]

1. Abrams SA: **Calcium turnover and nutrition through the life cycle**. *The Proceedings of the Nutrition Society* 2001, **60**(2):283-289.

| **Reason for exclusion** |  | Wrong study design |
| --- | --- | --- |

2. Abrams SA: **Normal acquisition and loss of bone mass**. *Hormone research* 2003, **60 Suppl 3**:71-76.

| **Reason for exclusion** | Wrong study design |
| --- | --- |

3. Alexe D-M, Syridou G, Petridou ET: **Determinants of early life leptin levels and later life degenerative outcomes**. *Clinical medicine & research* 2006, **4**(4):326-335.

| **Reason for exclusion** | Wrong study design |
| --- | --- |

4. Anderson JJ: **The role of nutrition in the functioning of skeletal tissue**. *Nutrition reviews* 1992, **50**(12):388-394.

| **Reason for exclusion** | Wrong study design |
| --- | --- |

5. Anderson JJ: **Calcium, phosphorus and human bone development**. *The Journal of nutrition* 1996, **126**(4 Suppl):1153S-1158S.

| **Reason for exclusion** | Wrong study design |
| --- | --- |

6. Barrett H: **Vitamin D and pregnancy: An old problem revisited**. *Best practice & research Clinical endocrinology & metabolism* 2010, **24**(4):527-539.

| **Reason for exclusion** | Wrong study design |
| --- | --- |

7. Becroft DM: **Prenatal cytomegalovirus infection: epidemiology, pathology and pathogenesis**. *Perspectives in pediatric pathology* 1981, **6**:203-241.

| **Reason for exclusion** | Wrong study design |
| --- | --- |

8. Berry CL: **Drugs and the developing skeleton**. *Investigative & cell pathology* 1978, **1**(2):129-137.

| **Reason for exclusion** | Wrong study design |
| --- | --- |

9. Bianchi ML: **How to manage osteoporosis in children**. *Best practice & research Clinical rheumatology* 2005, **19**(6):991-1005.

| **Reason for exclusion** | Wrong study design |
| --- | --- |

10. Bonjour JP et al: **Protein intake and bone growth**. *Canadian journal of applied physiology = Revue canadienne de physiologie appliquee* 2001, **26 Suppl**:S153-166.

| **Reason for exclusion** | Wrong intervention |
| --- | --- |

11. Bonjour J-P: **Pubertal timing, bone acquisition, and risk of fracture throughout life**. *Endocrine reviews* 2014, **35**(5):820-847.

| **Reason for exclusion** | Wrong study design |
| --- | --- |

12. Bouchard C: **Long-term programming of body size**. *Nutrition reviews* 1996, **54**(2 Pt 2):S8-16.

| **Reason for exclusion** | Wrong study design |
| --- | --- |

13. Caballero B: **Early nutrition and risk of disease in the adult**. *Public health nutrition* 2001, **4**(6A):1335-1336.

| **Reason for exclusion** | Wrong study design |
| --- | --- |

14. Callréus M, McGuigan F, Åkesson K: **Birth weight is more important for peak bone mineral content than for bone density: the PEAK-25 study of 1,061 young adult women**. *Osteoporosis international : a journal established as result of cooperation between the European Foundation for Osteoporosis and the National Osteoporosis Foundation of the USA* 2013, **24**(4):1347-1355.

| **Reason for exclusion** | Wrong intervention |
| --- | --- |

15. Cheng X, Wang G, Lee KKH, Yang X: **Dexamethasone use during pregnancy: potential adverse effects on embryonic skeletogenesis**. *Current pharmaceutical design* 2014, **20**(34):5430-5437.

| **Reason for exclusion** | Wrong study design |
| --- | --- |

16. Christoffersen T *et al*: **The influence of birth weight and length on bone mineral density and content in adolescence: The Tromsø Study, Fit Futures**. *Archives of osteoporosis* 2017, **12**(1):54.

| **Reason for exclusion** | Wrong intervention |
| --- | --- |

17. Cole ZA et al: **Maternal dietary patterns during pregnancy and childhood bone mass: a longitudinal study**. *Journal of bone and mineral research : the official journal of the American Society for Bone and Mineral Research* 2009, **24**(4):663-668.

| **Reason for exclusion** | Wrong outcomes |
| --- | --- |

18. Cooper C et al: **Developmental origins of osteoporosis: the role of maternal nutrition**. *Advances in experimental medicine and biology* 2009, **646**:31-39.

| **Reason for exclusion** | Wrong study design |
| --- | --- |

19. Cooper C et al: **Growth and bone development**. *Nestle Nutrition workshop series Paediatric programme* 2008, **61**:53-68.

| **Reason for exclusion** | Wrong study design |
| --- | --- |

20. Cooper C *et al*: **Maternal gestational vitamin D supplementation and offspring bone health (MAVIDOS): a multicentre, double-blind, randomised placebo-controlled trial**. *The lancet Diabetes & endocrinology* 2016, **4**(5):393-402.

| **Reason for exclusion** | Wrong outcomes |
| --- | --- |

21. Cooper C et al: **Review: developmental origins of osteoporotic fracture**. *Osteoporosis international : a journal established as result of cooperation between the European Foundation for Osteoporosis and the National Osteoporosis Foundation of the USA* 2006, **17**(3):337-347.

| **Reason for exclusion** | Wrong study design |
| --- | --- |

22. Cranney A *et al*: **Effectiveness and safety of vitamin D in relation to bone health**. *Evidence report/technology assessment* 2007(158):1-235.

| **Reason for exclusion** | Wrong study design |
| --- | --- |

23. Cunningham MD: **Determination of fetal maturity in diabetic pregnancy**. *Clinical obstetrics and gynecology* 1981, **24**(1):73-89.

| **Reason for exclusion** | Wrong study design |
| --- | --- |

24. Curtis E, Cheah J, Harvey NC: **Prenatal nutritional influence on skeletal development**. *World review of nutrition and dietetics* 2013, **106**:46-51.

| **Reason for exclusion** | Wrong study design |
| --- | --- |

25. Curtis EM et al: **Prenatal calcium and vitamin D intake, and bone mass in later life**. *Current osteoporosis reports* 2014, **12**(2):194-204.

| **Reason for exclusion** | Wrong study design |
| --- | --- |

26. Cusack S, Cashman KD: **Impact of genetic variation on metabolic response of bone to diet**. *The Proceedings of the Nutrition Society* 2003, **62**(4):901-912.

| **Reason for exclusion** | Wrong study design |
| --- | --- |

27. Davies JH, Evans BAJ, Gregory JW: **Bone mass acquisition in healthy children**. *Archives of disease in childhood* 2005, **90**(4):373-378.

| **Reason for exclusion** | Wrong study design |
| --- | --- |

28. Dawson-Hughes B: **Calcium insufficiency and fracture risk**. *Osteoporosis international : a journal established as result of cooperation between the European Foundation for Osteoporosis and the National Osteoporosis Foundation of the USA* 1996, **6 Suppl 3**:37-41.

| **Reason for exclusion** | Wrong study design |
| --- | --- |

29. Dawson-Hughes B: **Calcium and protein in bone health**. *The Proceedings of the Nutrition Society* 2003, **62**(2):505-509.

| **Reason for exclusion** | Wrong study design |
| --- | --- |

30. Demicheli V et al: **Vaccines for preventing influenza in healthy adults**. *Cochrane Database of Systematic Reviews* 2018(2).

| **Reason for exclusion** | Wrong study design |
| --- | --- |

31. Dennison EM et al: **Programming of osteoporosis and impact on osteoporosis risk**. *Clinical obstetrics and gynecology* 2013, **56**(3):549-555.

| **Reason for exclusion** | Wrong study design |
| --- | --- |

32. De‐Regil LM et al: **Vitamin D supplementation for women during pregnancy**. *Cochrane Database of Systematic Reviews* 2016(1).

| **Reason for exclusion** | Wrong study design |
| --- | --- |

33. De‐Regil LM et al: **Effects and safety of periconceptional oral folate supplementation for preventing birth defects**. *Cochrane Database of Systematic Reviews* 2015(12).

| **Reason for exclusion** | Wrong study design |
| --- | --- |

34. Deruelle P, Coulon C: **The use of low-molecular-weight heparins in pregnancy--how safe are they?** *Current opinion in obstetrics & gynecology* 2007, **19**(6):573-577.

| **Reason for exclusion** | Wrong study design |
| --- | --- |

35. Devlin MJ, Bouxsein ML: **Influence of pre- and peri-natal nutrition on skeletal acquisition and maintenance**. *Bone* 2012, **50**(2):444-451.

| **Reason for exclusion** | Wrong study design |
| --- | --- |

36. DiGiovanna JJ: **Isotretinoin effects on bone**. *Journal of the American Academy of Dermatology* 2001, **45**(5):S176-182.

| **Reason for exclusion** | Wrong study design |
| --- | --- |

37. Diogenes MEL: **Calcium Plus Vitamin D Supplementation During the Third Trimester of Pregnancy in Adolescents Accustomed to Low Calcium Diets Does Not Affect Infant Bone Mass at Early Lactation in a Randomized Controlled Trial**. *The Journal of nutrition* 2015, **145**(7):1515-1523.

| **Reason for exclusion** | Wrong patient population |
| --- | --- |

38. Dodd JM et al: **Metformin for women who are overweight or obese during pregnancy for improving maternal and infant outcomes**. *Cochrane Database of Systematic Reviews* 2018(7).

| **Reason for exclusion** | Wrong study design |
| --- | --- |

39. Dodd JM et al: **Prenatal administration of progestogens for preventing spontaneous preterm birth in women with a multiple pregnancy**. *Cochrane Database of Systematic Reviews* 2017(10).

| **Reason for exclusion** | Wrong study design |
| --- | --- |

40. Dror DK: **Vitamin D status during pregnancy: maternal, fetal, and postnatal outcomes**. *Current opinion in obstetrics & gynecology* 2011, **23**(6):422-426.

| **Reason for exclusion** | Wrong study design |
| --- | --- |

41. Dror DK et al: **Evidence of associations between feto-maternal vitamin D status, cord parathyroid hormone and bone-specific alkaline phosphatase, and newborn whole body bone mineral content**. *Nutrients* 2012, **4**(2):68-77.

| **Reason for exclusion** | Wrong outcomes |
| --- | --- |

42. Eastell R, Lambert H: **Diet and healthy bones**. *Calcified tissue international* 2002, **70**(5):400-404.

| **Reason for exclusion** | Wrong study design |
| --- | --- |

43. Egerman RS: **The tetracyclines**. *Obstetrics and gynecology clinics of North America* 1992, **19**(3):551-561.

| **Reason for exclusion** | Wrong study design |
| --- | --- |

44. Elsori DH, Hammoud MS: **Vitamin D deficiency in mothers, neonates and children**. *The Journal of steroid biochemistry and molecular biology* 2018, **175**:195-199.

| **Reason for exclusion** | Wrong study design |
| --- | --- |

45. Esterly JR, Oppenheimer EH: **Intrauterine rubella infection**. *Perspectives in pediatric pathology* 1973, **1**:313-338.

| **Reason for exclusion** | Wrong study design |
| --- | --- |

46. Farquharson RG: **Heparin, osteoporosis and pregnancy**. *British journal of hospital medicine* 1997, **58**(5):205-207.

| **Reason for exclusion** | Wrong study design |
| --- | --- |

47. Fasouliotis SJ, Ezra Y, Schenker JG: **Gaucher's disease and pregnancy**. *American journal of perinatology* 1998, **15**(5):311-318.

| **Reason for exclusion** | Wrong study design |
| --- | --- |

48. Fewtrell M: **Early nutritional predictors of long-term bone health in preterm infants**. *Current opinion in clinical nutrition and metabolic care* 2011, **14**(3):297-301.

| **Reason for exclusion** | Wrong study design |
| --- | --- |

49. Fewtrell MS: **Does early nutrition program later bone health in preterm infants?** *The American journal of clinical nutrition* 2011, **94**(6 Suppl):1870S-1873S.

| **Reason for exclusion** | Wrong patient population |
| --- | --- |

50. Fewtrell MS et al: **Early diet and peak bone mass: 20 year follow-up of a randomized trial of early diet in infants born preterm**. *Bone* 2009, **45**(1):142-149.

| **Reason for exclusion** | Wrong intervention |
| --- | --- |

51. Flynn A: **The role of dietary calcium in bone health**. *The Proceedings of the Nutrition Society* 2003, **62**(4):851-858.

| **Reason for exclusion** | Wrong study design |
| --- | --- |

52. Fonseca L  *et al*: **Bone metabolism in fetuses of pregnant women exposed to single and multiple courses of corticosteroids**. *Obstetrics and gynecology* 2009, **114**(1):38-44.

| **Reason for exclusion** | Wrong outcomes |
| --- | --- |

53. Frost HM, Schonau E: **The "muscle-bone unit" in children and adolescents: a 2000 overview**. *Journal of pediatric endocrinology & metabolism : JPEM* 2000, **13**(6):571-590.

| **Reason for exclusion** | Wrong study design |
| --- | --- |

54. Fulkerson JA et al: **Bone outcomes and technical measurement issues of bone health among children and adolescents: considerations for nutrition and physical activity intervention trials**. *Osteoporosis international : a journal established as result of cooperation between the European Foundation for Osteoporosis and the National Osteoporosis Foundation of the USA* 2004, **15**(12):929-941.

| **Reason for exclusion** | Wrong study design |
| --- | --- |

55. Ganpule A *et al*: **Bone mass in Indian children--relationships to maternal nutritional status and diet during pregnancy: the Pune Maternal Nutrition Study**. *The Journal of clinical endocrinology and metabolism* 2006, **91**(8):2994-3001.

| **Reason for exclusion** | Wrong outcomes |
| --- | --- |

56. Gibb DM *et al*: **Pregnancy and infant outcomes among HIV-infected women taking long-term ART with and without tenofovir in the DART trial**. *PLoS medicine* 2012, **9**(5):e1001217-e1001217.

| **Reason for exclusion** | Wrong outcomes |
| --- | --- |

57. Godfrey K et al: **Neonatal bone mass: influence of parental birthweight, maternal smoking, body composition, and activity during pregnancy**. *Journal of bone and mineral research : the official journal of the American Society for Bone and Mineral Research* 2001, **16**(9):1694-1703.

| **Reason for exclusion** | Wrong outcomes |
| --- | --- |

58. Goodfellow LR, Earl S, Cooper C, Harvey NC: **Maternal diet, behaviour and offspring skeletal health**. *International journal of environmental research and public health* 2010, **7**(4):1760-1772.

| **Reason for exclusion** | Wrong study design |
| --- | --- |

59. Gordon CM et al: **Bone health in children and adolescents: a symposium at the annual meeting of the Pediatric Academic Societies/Lawson Wilkins Pediatric Endocrine Society, May 2003**. *Current problems in pediatric and adolescent health care* 2004, **34**(6):226-242.

| **Reason for exclusion** | Wrong study design |
| --- | --- |

60. Grant WB, Boucher BJ: **Requirements for Vitamin D across the life span**. *Biological research for nursing* 2011, **13**(2):120-133.

| **Reason for exclusion** | Wrong study design |
| --- | --- |

61. Gray TK, Lowe W, Lester GE: **Vitamin D and pregnancy: the maternal-fetal metabolism of vitamin D**. *Endocrine reviews* 1981, **2**(3):264-274.

| **Reason for exclusion** | Wrong study design |
| --- | --- |

62. Hacker AN et al: **Role of calcium during pregnancy: maternal and fetal needs**. *Nutrition reviews* 2012, **70**(7):397-409.

| **Reason for exclusion** | Wrong study design |
| --- | --- |

63. Hanieh S et al: **Maternal vitamin D status and infant outcomes in rural Vietnam: a prospective cohort study**. *PloS one* 2014, **9**(6):e99005-e99005.

| **Reason for exclusion** | Wrong outcomes |
| --- | --- |

64. Harding KB et al: **Iodine supplementation for women during the preconception, pregnancy and postpartum period**. *Cochrane Database of Systematic Reviews* 2017(3).

| **Reason for exclusion** | Wrong study design |
| --- | --- |

65. Harinarayan CV, Joshi SR: **Vitamin D status in India--its implications and remedial measures**. *The Journal of the Association of Physicians of India* 2009, **57**:40-48.

| **Reason for exclusion** | Wrong study design |
| --- | --- |

66. Hart R, Norman RJ: **The longer-term health outcomes for children born as a result of IVF treatment: Part I--General health outcomes**. *Human reproduction update* 2013, **19**(3):232-243.

| **Reason for exclusion** | Wrong study design |
| --- | --- |

67. Harvey N, Cooper C: **Determinants of fracture risk in osteoporosis**. *Current rheumatology reports* 2003, **5**(1):75-81.

| **Reason for exclusion** | Wrong study design |
| --- | --- |

68. Harvey N, Cooper C: **The developmental origins of osteoporotic fracture**. *The journal of the British Menopause Society* 2004, **10**(1):14-15,29.

| **Reason for exclusion** | Wrong study design |
| --- | --- |

69. Harvey N et al: **Osteoporosis: a lifecourse approach**. *Journal of bone and mineral research : the official journal of the American Society for Bone and Mineral Research* 2014, **29**(9):1917-1925.

| **Reason for exclusion** | Wrong study design |
| --- | --- |

70. Harvey NC *et al*: **Vitamin D supplementation in pregnancy: a systematic review**. *Health technology assessment (Winchester, England)* 2014, **18**(45):1-190.

| **Reason for exclusion** | Wrong study design |
| --- | --- |

71. Harvey NC et al: **Maternal predictors of neonatal bone size and geometry: the Southampton Women's Survey**. *Journal of developmental origins of health and disease* 2010, **1**(1):35-41.

| **Reason for exclusion** | Wrong outcomes |
| --- | --- |

72. Haust MD: **Maternal diabetes mellitus--effects on the fetus and placenta**. *Monographs in pathology* 1981(22):201-285.

| **Reason for exclusion** | Wrong study design |
| --- | --- |

73. Himes JH: **Bone growth and development in protein-calorie malnutrition**. *World review of nutrition and dietetics* 1978, **28**:143-187.

| **Reason for exclusion** | Wrong study design |
| --- | --- |

74. Hogler W: **Complications of vitamin D deficiency from the foetus to the infant: One cause, one prevention, but who's responsibility?** *Best practice & research Clinical endocrinology & metabolism* 2015, **29**(3):385-398.

| **Reason for exclusion** | Wrong study design |
| --- | --- |

75. Holroyd C et al: **Epigenetic influences in the developmental origins of osteoporosis**. *Osteoporosis international : a journal established as result of cooperation between the European Foundation for Osteoporosis and the National Osteoporosis Foundation of the USA* 2012, **23**(2):401-410.

| **Reason for exclusion** | Wrong study design |
| --- | --- |

76. Holroyd CR: **Placental Size Is Associated Differentially With Postnatal Bone Size and Density**. *Journal of bone and mineral research : the official journal of the American Society for Bone and Mineral Research* 2016, **31**(10):1855-1864.

| **Reason for exclusion** | Wrong intervention |
| --- | --- |

77. Ilich JZ, Kerstetter JE: **Nutrition in bone health revisited: a story beyond calcium**. *Journal of the American College of Nutrition* 2000, **19**(6):715-737.

| **Reason for exclusion** | Wrong study design |
| --- | --- |

78. Jameson S, Ursing I: **Low serum zinc concentrations in pregnancy, results of investigations and treatment**. *Acta medica Scandinavica Supplementum* 1976, **593**:50-64.

| **Reason for exclusion** | Wrong outcomes |
| --- | --- |

79. Janakiraman V et al: **Calcium supplements and bone resorption in pregnancy: a randomized crossover trial**. *American journal of preventive medicine* 2003, **24**(3):260-264.

| **Reason for exclusion** | Wrong outcomes |
| --- | --- |

80. Janssen NM, Genta MS: **The effects of immunosuppressive and anti-inflammatory medications on fertility, pregnancy, and lactation**. *Archives of internal medicine* 2000, **160**(5):610-619.

| **Reason for exclusion** | Wrong study design |
| --- | --- |

81. Jarjou LMA et al: **Effect of calcium supplementation in pregnancy on maternal bone outcomes in women with a low calcium intake**. *The American journal of clinical nutrition* 2010, **92**(2):450-457.

| **Reason for exclusion** | Wrong outcomes |
| --- | --- |

82. Jarjou LMA et al: **Randomized, placebo-controlled, calcium supplementation study in pregnant Gambian women: effects on breast-milk calcium concentrations and infant birth weight, growth, and bone mineral accretion in the first year of life**. *The American journal of clinical nutrition* 2006, **83**(3):657-666.

| **Reason for exclusion** | Wrong outcomes |
| --- | --- |

83. Javaid MK, Cooper C: **Prenatal and childhood influences on osteoporosis**. *Best practice & research Clinical endocrinology & metabolism* 2002, **16**(2):349-367.

| **Reason for exclusion** | Wrong study design |
| --- | --- |

84. Javaid MK et al: **Maternal vitamin D status during pregnancy and childhood bone mass at age 9 years: a longitudinal study**. *Lancet (London, England)* 2006, **367**(9504):36-43.

| **Reason for exclusion** | Wrong outcomes |
| --- | --- |

85. Jensen RB: **Fetal growth velocity, size in early life and adolescence, and prediction of bone mass: association to the GH-IGF axis**. *Journal of bone and mineral research : the official journal of the American Society for Bone and Mineral Research* 2008, **23**(3):439-446.

| **Reason for exclusion** | Wrong outcomes |
| --- | --- |

86. Jones G: **Early life nutrition and bone development in children**. *Nestle Nutrition workshop series Paediatric programme* 2011, **68**:226-227.

| **Reason for exclusion** | Wrong study design |
| --- | --- |

87. Jones G: **Maternal smoking during pregnancy, growth, and bone mass in prepubertal children**. *Journal of bone and mineral research : the official journal of the American Society for Bone and Mineral Research* 1999, **14**(1):146-151.

| **Reason for exclusion** | Wrong outcomes |
| --- | --- |

88. Karlsson MK: **Female reproductive history and the skeleton-a review**. *BJOG : an international journal of obstetrics and gynaecology* 2005, **112**(7):851-856.

| **Reason for exclusion** | Wrong study design |
| --- | --- |

89. Karras SN *et al*: **Maternal vitamin D status during pregnancy: the Mediterranean reality**. *European journal of clinical nutrition* 2014, **68**(8):864-869.

| **Reason for exclusion** | Wrong study design |
| --- | --- |

90. Karras SN et al: **Maternal vitamin D status in pregnancy and offspring bone development: the unmet needs of vitamin D era**. *Osteoporosis international: a journal established as result of cooperation between the European Foundation for Osteoporosis and the National Osteoporosis Foundation of the USA* 2014, **25**(3):795-805.

| **Reason for exclusion** | Wrong study design |
| --- | --- |

91. Keats EC et al: **Multiple‐micronutrient supplementation for women during pregnancy**. *Cochrane Database of Systematic Reviews* 2019(3).

| **Reason for exclusion** | Wrong study design |
| --- | --- |

92. Khovidhunkit W, Epstein S: **Osteoporosis in pregnancy**. *Osteoporosis international : a journal established as result of cooperation between the European Foundation for Osteoporosis and the National Osteoporosis Foundation of the USA* 1996, **6**(5):345-354.

| **Reason for exclusion** | Wrong study design |
| --- | --- |

93. Kohlmeier L, Marcus R: **Calcium disorders of pregnancy**. *Endocrinology and metabolism clinics of North America* 1995, **24**(1):15-39.

| **Reason for exclusion** | Wrong study design |
| --- | --- |

94. Koletzko B et al: **Growth, development and differentiation: a functional food science approach**. *The British journal of nutrition* 1998, **80 Suppl 1**:S5-45.

| **Reason for exclusion** | Wrong study design |
| --- | --- |

95. Koo WW et al: **Maternal calcium supplementation and fetal bone mineralization**. *Obstetrics and gynecology* 1999, **94**(4):577-582.

| **Reason for exclusion** | Wrong outcomes |
| --- | --- |

96. Kovacs CS: **Vitamin D in pregnancy and lactation: maternal, fetal, and neonatal outcomes from human and animal studies**. *The American journal of clinical nutrition* 2008, **88**(2):520S-528S.

| **Reason for exclusion** | Wrong study design |
| --- | --- |

97. Krebs NF: **Bioavailability of dietary supplements and impact of physiologic state: infants, children and adolescents**. *The Journal of nutrition* 2001, **131**(4 Suppl):1351S-1354S.

| **Reason for exclusion** | Wrong study design |
| --- | --- |

98. Lalor JG et al: **Biophysical profile for fetal assessment in high risk pregnancies**. *Cochrane Database of Systematic Reviews* 2008(1).

| **Reason for exclusion** | Wrong study design |
| --- | --- |

99. Lamberg-Allardt C: **Vitamin D in children and adolescents**. *Scandinavian journal of clinical and laboratory investigation Supplementum* 2012, **243**:124-128.

| **Reason for exclusion** | Wrong study design |
| --- | --- |

100. Lassi ZS et al: **Folic acid supplementation during pregnancy for maternal health and pregnancy outcomes**. *The Cochrane database of systematic reviews* 2013(3):CD006896-CD006896.

| **Reason for exclusion** | Wrong study design |
| --- | --- |

101. Lawlor DA et al: **Association of maternal vitamin D status during pregnancy with bone-mineral content in offspring: a prospective cohort study**. *Lancet (London, England)* 2013, **381**(9884):2176-2183.

| **Reason for exclusion** | Wrong outcomes |
| --- | --- |

102. Le Templier G, Rodger MA: **Heparin-induced osteoporosis and pregnancy**. *Current opinion in pulmonary medicine* 2008, **14**(5):403-407.

| **Reason for exclusion** | Wrong study design |
| --- | --- |

103. Leonard MB, Zemel BS: **Current concepts in pediatric bone disease**. *Pediatric clinics of North America* 2002, **49**(1):143-173.

| **Reason for exclusion** | Wrong study design |
| --- | --- |

104. Lerchbaum E, Rabe T: **Vitamin D and female fertility**. *Current opinion in obstetrics & gynecology* 2014, **26**(3):145-150.

| **Reason for exclusion** | Wrong study design |
| --- | --- |

105. Leunissen RW et al: **Influence of birth size and body composition on bone mineral density in early adulthood: the PROGRAM study**. *Clinical endocrinology* 2008, **69**(3):386-392.

| **Reason for exclusion** | Wrong intervention |
| --- | --- |

106. Levis S, Lagari VS: **The role of diet in osteoporosis prevention and management**. *Current osteoporosis reports* 2012, **10**(4):296-302.

| **Reason for exclusion** | Wrong study design |
| --- | --- |

107. Lewin S *et al*: **Lay health workers in primary and community health care for maternal and child health and the management of infectious diseases**. *Cochrane Database of Systematic Reviews* 2010(3).

| **Reason for exclusion** | Wrong study design |
| --- | --- |

108. Lewis RD, Laing EM: **Conflicting reports on vitamin D supplementation: Evidence from randomized controlled trials**. *Molecular and cellular endocrinology* 2015, **410**:11-18.

| **Reason for exclusion** | Wrong study design |
| --- | --- |

109. Lewis S et al: **Vitamin D deficiency and pregnancy: from preconception to birth**. *Molecular nutrition & food research* 2010, **54**(8):1092-1102.

| **Reason for exclusion** | Wrong study design |
| --- | --- |

110. Li Y et al: **Identification of risk factors affecting bone formation in gradual ulnar lengthening in children with hereditary multiple exostoses: A retrospective study**. *Medicine* 2019, **98**(5):e14280-e14280.

| **Reason for exclusion** | Wrong outcomes |
| --- | --- |

111. Lin S et al: **Maternal asthma medication use and the risk of selected birth defects**. *Pediatrics* 2012, **129**(2):e317-324.

| **Reason for exclusion** | Wrong outcomes |
| --- | --- |

112. Liporace J, D'Abreu A: **Epilepsy and women's health: family planning, bone health, menopause, and menstrual-related seizures**. *Mayo Clinic proceedings* 2003, **78**(4):497-506.

| **Reason for exclusion** | Wrong study design |
| --- | --- |

113. Litmanovitz I et al: **High Beta-palmitate formula and bone strength in term infants: a randomized, double-blind, controlled trial**. *Calcified tissue international* 2013, **92**(1):35-41.

| **Reason for exclusion** | Wrong outcomes |
| --- | --- |

114. Liu X et al: **Reduced maternal calcium intake through nutrition and supplementation is associated with adverse conditions for both the women and their infants in a Chinese population**. *Medicine* 2017, **96**(18):e6609-e6609.

| **Reason for exclusion** | Wrong outcomes |
| --- | --- |

115. Liu Z et al: **Effect of milk and calcium supplementation on bone density and bone turnover in pregnant Chinese women: a randomized controlled trail**. *Archives of gynecology and obstetrics* 2011, **283**(2):205-211.

| **Reason for exclusion** | Wrong outcomes |
| --- | --- |

116. Lloyd ME et al: **The effects of methotrexate on pregnancy, fertility and lactation**. *QJM : monthly journal of the Association of Physicians* 1999, **92**(10):551-563.

| **Reason for exclusion** | Wrong study design |
| --- | --- |

117. Lloyd T, Taylor DS: **Calcium intake and peak bone mass**. *Journal of the American Medical Women's Association (1972)* 2001, **56**(2):49-52,72.

| **Reason for exclusion** | Wrong study design |
| --- | --- |

118. Loro ML et al: **Early identification of children predisposed to low peak bone mass and osteoporosis later in life**. *The Journal of clinical endocrinology and metabolism* 2000, **85**(10):3908-3918.

| **Reason for exclusion** | Wrong patient population |
| --- | --- |

119. Lowdon J: **Getting bone health right from the start! Pregnancy, lactation and weaning**. *The journal of family health care* 2008, **18**(4):137-141.

| **Reason for exclusion** | Wrong study design |
| --- | --- |

120. Lu M et al: **Association between vitamin D status and the risk of gestational diabetes mellitus: a meta-analysis**. *Archives of gynecology and obstetrics* 2016, **293**(5):959-966.

| **Reason for exclusion** | Wrong study design |
| --- | --- |

121. Lucas A: **Programming by early nutrition: an experimental approach**. *The Journal of nutrition* 1998, **128**(2 Suppl):401S-406S.

| **Reason for exclusion** | Wrong study design |
| --- | --- |

122. Lucas P et al: **Financial benefits for child health and well‐being in low income or socially disadvantaged families in developed world countries**. *Cochrane Database of Systematic Reviews* 2008(2).

| **Reason for exclusion** | Wrong study design |
| --- | --- |

123. Lui JC et al: **Regulation of body growth**. *Current opinion in pediatrics* 2015, **27**(4):502-510.

| **Reason for exclusion** | Wrong study design |
| --- | --- |

124. Lunt H: **Women and diabetes**. *Diabetic medicine : a journal of the British Diabetic Association* 1996, **13**(12):1009-1016.

| **Reason for exclusion** | Wrong study design |
| --- | --- |

125. Lutwak L: **Continuing need for dietary calcium throughout life**. *Geriatrics* 1974, **29**(5):171-174 passim.

| **Reason for exclusion** | Wrong study design |
| --- | --- |

126. Mahran A et al: **The effect of sacral neuromodulation on pregnancy: a systematic review**. *International urogynecology journal* 2017, **28**(9):1357-1365.

| **Reason for exclusion** | Wrong study design |
| --- | --- |

127. Makrides M et al: **Magnesium supplementation in pregnancy**. *Cochrane Database of Systematic Reviews* 2014(4).

| **Reason for exclusion** | Wrong study design |
| --- | --- |

128. Mangano KM et al: **Bone Mineral Density and Protein-Derived Food Clusters from the Framingham Offspring Study**. *Journal of the Academy of Nutrition and Dietetics* 2015, **115**(10):1605-1613.e1601.

| **Reason for exclusion** | Wrong patient population |
| --- | --- |

129. Marie PJ: **Strontium ranelate: a physiological approach for optimizing bone formation and resorption**. *Bone* 2006, **38**(2 Suppl 1):S10-14.

| **Reason for exclusion** | Wrong study design |
| --- | --- |

130. Marles SL, Reed M, Evans JA: **Humeroradial synostosis, ulnar aplasia and oligodactyly, with contralateral amelia, in a child with prenatal cocaine exposure**. *American journal of medical genetics Part A* 2003, **116A**(1):85-89.

| **Reason for exclusion** | Wrong study design |
| --- | --- |

131. Martí‐Carvajal AJ et al: **Medical treatments for idiopathic thrombocytopenic purpura during pregnancy**. *Cochrane Database of Systematic Reviews* 2009(4).

| **Reason for exclusion** | Wrong study design |
| --- | --- |

132. Mateussi MV et al: **What do Cochrane systematic reviews say about interventions for vitamin D supplementation?** *Sao Paulo medical journal = Revista paulista de medicina* 2017, **135**(5):497-507.

| **Reason for exclusion** | Wrong study design |
| --- | --- |

133. Matkovic V, Ilich JZ: **Calcium requirements for growth: are current recommendations adequate?** *Nutrition reviews* 1993, **51**(6):171-180.

| **Reason for exclusion** | Wrong study design |
| --- | --- |

134. Matsuzaki M *et al*: **Life-course determinants of bone mass in young adults from a transitional rural community in India: the Andhra Pradesh Children and Parents Study (APCAPS)**. *The American journal of clinical nutrition* 2014, **99**(6):1450-1459.

| **Reason for exclusion** | Wrong patient population |
| --- | --- |

135. McCauley ME et al: **Vitamin A supplementation during pregnancy for maternal and newborn outcomes**. *Cochrane Database of Systematic Reviews* 2015(10).

| **Reason for exclusion** | Wrong study design |
| --- | --- |

136. McClung JP, Gaffney-Stomberg E: **Optimizing Performance, Health, and Well-being: Nutritional Factors**. *Military medicine* 2016, **181**(1 Suppl):86-91.

| **Reason for exclusion** | Wrong study design |
| --- | --- |

137. McNamara HC et al: **Different treatment regimens of magnesium sulphate for tocolysis in women in preterm labour**. *The Cochrane database of systematic reviews* 2015(12):CD011200-CD011200.

| **Reason for exclusion** | Wrong study design |
| --- | --- |

138. Meher S et al: **Bed rest with or without hospitalisation for hypertension during pregnancy**. *Cochrane Database of Systematic Reviews* 2005(4).

| **Reason for exclusion** | Wrong study design |
| --- | --- |

139. Melnik BC et al: **Milk consumption during pregnancy increases birth weight, a risk factor for the development of diseases of civilization**. *Journal of translational medicine* 2015, **13**:13-13.

| **Reason for exclusion** | Wrong study design |
| --- | --- |

140. Micklesfield LK et al: **Early life and current determinants of bone in South African children of mixed ancestral origin**. *Annals of human biology* 2007, **34**(6):647-655.

| **Reason for exclusion** | Wrong outcomes |
| --- | --- |

141. Middleton P et al: **Omega‐3 fatty acid addition during pregnancy**. *Cochrane Database of Systematic Reviews* 2018(11).

| **Reason for exclusion** | Wrong study design |
| --- | --- |

142. Miller GD et al: **Age considerations in nutrient needs for bone health**. *Journal of the American College of Nutrition* 1996, **15**(6):553-555.

| **Reason for exclusion** | Wrong study design |
| --- | --- |

143. Miller M et al: **Overrepresentation of multiple birth pregnancies in young infants with four metabolic bone disorders: further evidence that fetal bone loading is a critical determinant of fetal and young infant bone strength**. *Osteoporosis international : a journal established as result of cooperation between the European Foundation for Osteoporosis and the National Osteoporosis Foundation of the USA* 2014, **25**(7):1861-1873.

| **Reason for exclusion** | Wrong outcomes |
| --- | --- |

144. Millward DJ: **Optimal intakes of protein in the human diet**. *The Proceedings of the Nutrition Society* 1999, **58**(2):403-413.

| **Reason for exclusion** | Wrong study design |
| --- | --- |

145. Milo G et al: **Duration of antibacterial treatment for uncomplicated urinary tract infection in women**. *Cochrane Database of Systematic Reviews* 2005(2).

| **Reason for exclusion** | Wrong study design |
| --- | --- |

146. Mirza FG et al: **Trauma in pregnancy: a systematic approach**. *American journal of perinatology* 2010, **27**(7):579-586.

| **Reason for exclusion** | Wrong study design |
| --- | --- |

147. Mitchell JA *et al*: **Genetics of Bone Mass in Childhood and Adolescence: Effects of Sex and Maturation Interactions**. *Journal of bone and mineral research : the official journal of the American Society for Bone and Mineral Research* 2015, **30**(9):1676-1683.

| **Reason for exclusion** | Wrong intervention |
| --- | --- |

148. Mitchell PJ et al: **Life-course approach to nutrition**. *Osteoporosis international : a journal established as result of cooperation between the European Foundation for Osteoporosis and the National Osteoporosis Foundation of the USA* 2015, **26**(12):2723-2742.

| **Reason for exclusion** | Wrong study design |
| --- | --- |

149. Mofenson LM et al: **Tenofovir disoproxil fumarate safety for women and their infants during pregnancy and breastfeeding**. *AIDS (London, England)* 2017, **31**(2):213-232.

| **Reason for exclusion** | Wrong study design |
| --- | --- |

150. Molgaard C et al: **Does vitamin D supplementation of healthy Danish Caucasian girls affect bone turnover and bone mineralization?** *Bone* 2010, **46**(2):432-439.

| **Reason for exclusion** | Wrong intervention |
| --- | --- |

151. Montouris G: **Importance of monotherapy in women across the reproductive cycle**. *Neurology* 2007, **69**(24 Suppl 3):S10-16.

| **Reason for exclusion** | Wrong study design |
| --- | --- |

152. Mook-Kanamori DO et al: **Heritability estimates of body size in fetal life and early childhood**. *PloS one* 2012, **7**(7):e39901-e39901.

| **Reason for exclusion** | Wrong intervention |
| --- | --- |

153. Moon RJ et al: **ENDOCRINOLOGY IN PREGNANCY: Influence of maternal vitamin D status on obstetric outcomes and the fetal skeleton**. *European journal of endocrinology* 2015, **173**(2):R69-83.

| **Reason for exclusion** | Wrong study design |
| --- | --- |

154. Mora S, Gilsanz V: **Establishment of peak bone mass**. *Endocrinology and metabolism clinics of North America* 2003, **32**(1):39-63.

| **Reason for exclusion** | Wrong study design |
| --- | --- |

155. Morrell MJ: **The new antiepileptic drugs and women: efficacy, reproductive health, pregnancy, and fetal outcome**. *Epilepsia* 1996, **37 Suppl 6**:S34-44.

| **Reason for exclusion** | Wrong study design |
| --- | --- |

156. Morrell MJ: **Reproductive and metabolic disorders in women with epilepsy**. *Epilepsia* 2003, **44 Suppl 4**:11-20.

| **Reason for exclusion** | Wrong study design |
| --- | --- |

157. Morris HA et al: **Experimental evidence for the effects of calcium and vitamin D on bone: a review**. *Nutrients* 2010, **2**(9):1026-1035.

| **Reason for exclusion** | Wrong study design |
| --- | --- |

158. Mousa A et al: **Vitamin D in Reproductive Health and Pregnancy**. *Seminars in reproductive medicine* 2016, **34**(2):e1-13.

| **Reason for exclusion** | Wrong study design |
| --- | --- |

159. Mueller BA et al: **Pregnancy outcomes in female childhood and adolescent cancer survivors: a linked cancer-birth registry analysis**. *Archives of pediatrics & adolescent medicine* 2009, **163**(10):879-886.

| **Reason for exclusion** | Wrong outcomes |
| --- | --- |

160. Mughal MZ et al: **Body composition and bone status of children born to mothers with type 1 diabetes mellitus**. *Archives of disease in childhood* 2010, **95**(4):281-285.

| **Reason for exclusion** | Wrong outcomes |
| --- | --- |

161. Mughal MZ, Khadilkar AV: **The accrual of bone mass during childhood and puberty**. *Current opinion in endocrinology, diabetes, and obesity* 2011, **18**(1):28-32.

| **Reason for exclusion** | Wrong study design |
| --- | --- |

162. Nachega JB et al: **Safety of Tenofovir Disoproxil Fumarate-Based Antiretroviral Therapy Regimens in Pregnancy for HIV-Infected Women and Their Infants: A Systematic Review and Meta-Analysis**. *Journal of acquired immune deficiency syndromes (1999)* 2017, **76**(1):1-12.

| **Reason for exclusion** | Wrong study design |
| --- | --- |

163. Normando P et al: **Calcium plus vitamin D supplementation during pregnancy interacts with polymorphisms in the promoter region of the VDR gene to affect postpartum bone mass of Brazilian adolescent mothers: A randomized controlled trial**. *Nutrition (Burbank, Los Angeles County, Calif)* 2016, **32**(10):1068-1074.

| **Reason for exclusion** | Wrong outcomes |
| --- | --- |

164. O'Callaghan KM, Kiely ME: **Ethnic disparities in the dietary requirement for vitamin D during pregnancy: considerations for nutrition policy and research**. *The Proceedings of the Nutrition Society* 2018, **77**(2):164-173.

| **Reason for exclusion** | Wrong study design |
| --- | --- |

165. Olausson H et al: **Calcium economy in human pregnancy and lactation**. *Nutrition research reviews* 2012, **25**(1):40-67.

| **Reason for exclusion** | Wrong study design |
| --- | --- |

166. Ota E et al: **Zinc supplementation for improving pregnancy and infant outcome**. *Cochrane Database of Systematic Reviews* 2015(2).

| **Reason for exclusion** | Wrong study design |
| --- | --- |

167. Othman M et al: **Probiotics for preventing preterm labour**. *Cochrane Database of Systematic Reviews* 2007(1).

| **Reason for exclusion** | Wrong study design |
| --- | --- |

168. Othman M et al: **Non‐opioid drugs for pain management in labour**. *Cochrane Database of Systematic Reviews* 2012(7).

| **Reason for exclusion** | Wrong study design |
| --- | --- |

169. Parkes I et al: **Parathyroid and calcium metabolism disorders during pregnancy**. *Gynecological endocrinology : the official journal of the International Society of Gynecological Endocrinology* 2013, **29**(6):515-519.

| **Reason for exclusion** | Wrong study design |
| --- | --- |

170. Pawley N, Bishop NJ: **Prenatal and infant predictors of bone health: the influence of vitamin D**. *The American journal of clinical nutrition* 2004, **80**(6 Suppl):1748S-1751S.

| **Reason for exclusion** | Wrong study design |
| --- | --- |

171. Peña‐Rosas JP et al: **Daily oral iron supplementation during pregnancy**. *Cochrane Database of Systematic Reviews* 2015(7).

| **Reason for exclusion** | Wrong study design |
| --- | --- |

172. Peña‐Rosas JP et al: **Intermittent oral iron supplementation during pregnancy**. *Cochrane Database of Systematic Reviews* 2015(10).

| **Reason for exclusion** | Wrong study design |
| --- | --- |

173. Petersen SB et al: **Maternal vitamin D status and offspring bone fractures: prospective study over two decades in Aarhus City, Denmark**. *PloS one* 2014, **9**(12):e114334.

| **Reason for exclusion** | Wrong outcomes |
| --- | --- |

174. Peyron F et al: **Treatments for toxoplasmosis in pregnancy**. *Cochrane Database of Systematic Reviews* 1999(3).

| **Reason for exclusion** | Wrong study design |
| --- | --- |

175. Pieltain C et al: **Prematurity and bone health**. *World review of nutrition and dietetics* 2013, **106**:181-188.

| **Reason for exclusion** | Wrong study design |
| --- | --- |

176. Pludowski P *et al*: **Vitamin D effects on musculoskeletal health, immunity, autoimmunity, cardiovascular disease, cancer, fertility, pregnancy, dementia and mortality-a review of recent evidence**. *Autoimmunity reviews* 2013, **12**(10):976-989.

| **Reason for exclusion** | Wrong study design |
| --- | --- |

177. Porthouse J et al: **Risk factors for fracture in a UK population: a prospective cohort study**. *QJM : monthly journal of the Association of Physicians* 2004, **97**(9):569-574.

| **Reason for exclusion** | Wrong patient population |
| --- | --- |

178. Prentice A: **Maternal calcium metabolism and bone mineral status**. *The American journal of clinical nutrition* 2000, **71**(5 Suppl):1312S-1316S.

| **Reason for exclusion** | Wrong study design |
| --- | --- |

179. Prentice A: **Calcium in pregnancy and lactation**. *Annual review of nutrition* 2000, **20**:249-272.

| **Reason for exclusion** | Wrong study design |
| --- | --- |

180. Prentice A: **Micronutrients and the bone mineral content of the mother, fetus and newborn**. *The Journal of nutrition* 2003, **133**(5 Suppl 2):1693S-1699S.

| **Reason for exclusion** | Wrong study design |
| --- | --- |

181. Prentice A: **Milk intake, calcium and vitamin D in pregnancy and lactation: effects on maternal, fetal and infant bone in low- and high-income countries**. *Nestle Nutrition workshop series Paediatric programme* 2011, **67**:1-15.

| **Reason for exclusion** | Wrong study design |
| --- | --- |

182. Prentice A et al: **Maternal plasma 25-hydroxyvitamin D concentration and birthweight, growth and bone mineral accretion of Gambian infants**. *Acta paediatrica (Oslo, Norway : 1992)* 2009, **98**(8):1360-1362.

| **Reason for exclusion** | Wrong outcomes |
| --- | --- |

183. Prentice A et al: **Nutrition and bone growth and development**. *The Proceedings of the Nutrition Society* 2006, **65**(4):348-360.

| **Reason for exclusion** | Wrong study design |
| --- | --- |

184. Prick BW et al: **Maternal phenylketonuria and hyperphenylalaninemia in pregnancy: pregnancy complications and neonatal sequelae in untreated and treated pregnancies**. *The American journal of clinical nutrition* 2012, **95**(2):374-382.

| **Reason for exclusion** | Wrong study design |
| --- | --- |

185. Principi N et al: **Implications of maternal vitamin D deficiency for the fetus, the neonate and the young infant**. *European journal of nutrition* 2013, **52**(3):859-867.

| **Reason for exclusion** | Wrong study design |
| --- | --- |

186. Puthanakit T, Siberry GK: **Bone health in children and adolescents with perinatal HIV infection**. *Journal of the International AIDS Society* 2013, **16**:18575-18575.

| **Reason for exclusion** | Wrong study design |
| --- | --- |

187. Ralston SH: **What determines peak bone mass and bone loss?** *Bailliere's clinical rheumatology* 1997, **11**(3):479-494.

| **Reason for exclusion** | Wrong study design |
| --- | --- |

188. Rasmussen C: **Hyperprolactinaemia--a clinical study with special reference to long-term follow-up, treatment with dopamine agonists, and pregnancy**. *Upsala journal of medical sciences* 1990, **95**(1):1-29.

| **Reason for exclusion** | Wrong outcomes |
| --- | --- |

189. Rautiainen S et al: **Dietary supplements and disease prevention - a global overview**. *Nature reviews Endocrinology* 2016, **12**(7):407-420.

| **Reason for exclusion** | Wrong study design |
| --- | --- |

190. Rawlinson W, Scott G: **Cytomegalovirus. A common virus causing serious disease**. *Australian family physician* 2003, **32**(10):789-793.

| **Reason for exclusion** | Wrong study design |
| --- | --- |

191. Reece AS: **Chronic toxicology of cannabis**. *Clinical toxicology (Philadelphia, Pa)* 2009, **47**(6):517-524.

| **Reason for exclusion** | Wrong study design |
| --- | --- |

192. Regev RH et al: **Bone speed of sound in infants of mothers with gestational diabetes mellitus**. *Journal of pediatric endocrinology & metabolism : JPEM* 2004, **17**(8):1083-1088.

| **Reason for exclusion** | Wrong outcomes |
| --- | --- |

193. Reitnauer P et al: **Prenatal exposure to disulfiram implicated in the cause of malformations in discordant monozygotic twins**. *Teratology* 1997, **56**(6):358-362.

| **Reason for exclusion** | Wrong study design |
| --- | --- |

194. Rengasamy P: **Congenital Malformations Attributed to Prenatal Exposure to Cyclophosphamide**. *Anti-cancer agents in medicinal chemistry* 2017, **17**(9):1211-1227.

| **Reason for exclusion** | Wrong study design |
| --- | --- |

195. Retnakaran A, Retnakaran R: **Adiponectin in pregnancy: implications for health and disease**. *Current medicinal chemistry* 2012, **19**(32):5444-5450.

| **Reason for exclusion** | Wrong study design |
| --- | --- |

196. Reveiz L et al: **Treatments for iron‐deficiency anaemia in pregnancy**. *Cochrane Database of Systematic Reviews* 2011(10).

| **Reason for exclusion** | Wrong study design |
| --- | --- |

197. Rizzoli R: **Nutritional aspects of bone health**. *Best practice & research Clinical endocrinology & metabolism* 2014, **28**(6):795-808.

| **Reason for exclusion** | Wrong study design |
| --- | --- |

198. Rodger MA et al: **Long-term dalteparin in pregnancy not associated with a decrease in bone mineral density: substudy of a randomized controlled trial**. *Journal of thrombosis and haemostasis : JTH* 2007, **5**(8):1600-1606.

| **Reason for exclusion** | Wrong outcomes |
| --- | --- |

199. Rodriguez-Pinilla E et al: **Prenatal exposure to valproic acid during pregnancy and limb deficiencies: a case-control study**. *American journal of medical genetics* 2000, **90**(5):376-381.

| **Reason for exclusion** | Wrong outcomes |
| --- | --- |

200. Root AW: **Bone strength and the adolescent**. *Adolescent medicine (Philadelphia, Pa)* 2002, **13**(1):53-72, vi.

| **Reason for exclusion** | Wrong study design |
| --- | --- |

201. Rosnes JS et al: **Gaucher's disease in pregnancy**. *Obstetrical & gynecological survey* 1996, **51**(9):549-558.

| **Reason for exclusion** | Wrong study design |
| --- | --- |

202. Ruiz-Irastorza G et al: **Heparin and osteoporosis during pregnancy: 2002 update**. *Lupus* 2002, **11**(10):680-682.

| **Reason for exclusion** | Wrong study design |
| --- | --- |

203. Ruiz-Irastorza G et al: **Lupus pregnancy: is heparin a risk factor for osteoporosis?** *Lupus* 2001, **10**(9):597-600.

| **Reason for exclusion** | Wrong study design |
| --- | --- |

204. Rumbold A et al: **Vitamin C supplementation in pregnancy**. *Cochrane Database of Systematic Reviews* 2015(9).

| **Reason for exclusion** | Wrong study design |
| --- | --- |

205. Rumrich IK et al: **Maternal Smoking and the Risk of Cancer in Early Life - A Meta-Analysis**. *PloS one* 2016, **11**(11):e0165040-e0165040.

| **Reason for exclusion** | Wrong study design |
| --- | --- |

206. Ryan S: **Bone mineralization and growth**. *European journal of clinical nutrition* 1992, **46 Suppl 4**:S41-44.

| **Reason for exclusion** | Wrong study design |
| --- | --- |

207. Saggese G et al: **Puberty and bone development**. *Best practice & research Clinical endocrinology & metabolism* 2002, **16**(1):53-64.

| **Reason for exclusion** | Wrong study design |
| --- | --- |

208. Sahoo SK et al: **Maternal vitamin D supplementation in pregnancy and offspring outcomes: a double-blind randomized placebo-controlled trial**. *Journal of bone and mineral metabolism* 2017, **35**(4):464-471.

| **Reason for exclusion** | Wrong outcomes |
| --- | --- |

209. Salam RA et al: **Pyridoxine (vitamin B6) supplementation during pregnancy or labour for maternal and neonatal outcomes**. *Cochrane Database of Systematic Reviews* 2015(6).

| **Reason for exclusion** | Wrong study design |
| --- | --- |

210. Salmasi G et al: **Environmental tobacco smoke exposure and perinatal outcomes: a systematic review and meta-analyses**. *Acta obstetricia et gynecologica Scandinavica* 2010, **89**(4):423-441.

| **Reason for exclusion** | Wrong study design |
| --- | --- |

211. Sayer AA, Cooper C: **Early diet and growth: impact on ageing**. *The Proceedings of the Nutrition Society* 2002, **61**(1):79-85.

| **Reason for exclusion** | Wrong study design |
| --- | --- |

212. Sayers A, Tobias JH: **Estimated maternal ultraviolet B exposure levels in pregnancy influence skeletal development of the child**. *The Journal of clinical endocrinology and metabolism* 2009, **94**(3):765-771.

| **Reason for exclusion** | Wrong outcomes |
| --- | --- |

213. Schlussel MM et al: **Birth weight and adult bone mass: a systematic literature review**. *Osteoporosis international : a journal established as result of cooperation between the European Foundation for Osteoporosis and the National Osteoporosis Foundation of the USA* 2010, **21**(12):1981-1991.

| **Reason for exclusion** | Wrong study design |
| --- | --- |

214. Sopher AB et al: **An update on childhood bone health: mineral accrual, assessment and treatment**. *Current opinion in endocrinology, diabetes, and obesity* 2015, **22**(1):35-40.

| **Reason for exclusion** | Wrong study design |
| --- | --- |

215. Specker B: **Vitamin D requirements during pregnancy**. *The American journal of clinical nutrition* 2004, **80**(6 Suppl):1740S-1747S.

| **Reason for exclusion** | Wrong study design |
| --- | --- |

216. Thomas M, Weisman SM: **Calcium supplementation during pregnancy and lactation: effects on the mother and the fetus**. *American journal of obstetrics and gynecology* 2006, **194**(4):937-945.

| **Reason for exclusion** | Wrong study design |
| --- | --- |

217. Thorne-Lyman A, Fawzi WW: **Vitamin D during pregnancy and maternal, neonatal and infant health outcomes: a systematic review and meta-analysis**. *Paediatric and perinatal epidemiology* 2012, **26 Suppl 1**:75-90.

| **Reason for exclusion** | Wrong study design |
| --- | --- |

218. Uneke CJ: **Impact of placental Plasmodium falciparum malaria on pregnancy and perinatal outcome in sub-Saharan Africa: part III: placental malaria, maternal health, and public health**. *The Yale journal of biology and medicine* 2008, **81**(1):1-7.

| **Reason for exclusion** | Wrong study design |
| --- | --- |

219. Urrutia RP, Thorp JM: **Vitamin D in pregnancy: current concepts**. *Current opinion in obstetrics & gynecology* 2012, **24**(2):57-64.

| **Reason for exclusion** | Wrong study design |
| --- | --- |

220. Victora CG et al: **Maternal and child undernutrition: consequences for adult health and human capital**. *Lancet (London, England)* 2008, **371**(9609):340-357.

| **Reason for exclusion** | Wrong study design |
| --- | --- |

221. Vidulich L et al: **Infant programming of bone size and bone mass in 10-year-old black and white South African children**. *Paediatric and perinatal epidemiology* 2007, **21**(4):354-362.

| **Reason for exclusion** | Wrong outcomes |
| --- | --- |

222. Viljakainen HT: **Factors influencing bone mass accrual: focus on nutritional aspects**. *The Proceedings of the Nutrition Society* 2016, **75**(3):415-419.

| **Reason for exclusion** | Wrong study design |
| --- | --- |

223. Viljakainen HT et al: **Maternal vitamin D status affects bone growth in early childhood--a prospective cohort study**. *Osteoporosis international : a journal established as result of cooperation between the European Foundation for Osteoporosis and the National Osteoporosis Foundation of the USA* 2011, **22**(3):883-891.

| **Reason for exclusion** | Wrong outcomes |
| --- | --- |

224. Viljakainen HT et al: **Maternal vitamin D status determines bone variables in the newborn**. *The Journal of clinical endocrinology and metabolism* 2010, **95**(4):1749-1757.

| **Reason for exclusion** | Wrong outcomes |
| --- | --- |

225. Vinding RK et al: **Effect of fish oil supplementation in pregnancy on bone, lean, and fat mass at six years: randomised clinical trial**. *BMJ (Clinical research ed)* 2018, **362**:k3312-k3312.

| **Reason for exclusion** | Wrong outcomes |
| --- | --- |

226. Wagner CL et al: **Vitamin D and its role during pregnancy in attaining optimal health of mother and fetus**. *Nutrients* 2012, **4**(3):208-230.

| **Reason for exclusion** | Wrong study design |
| --- | --- |

227. Wikoff D *et al*: **Systematic review of the potential adverse effects of caffeine consumption in healthy adults, pregnant women, adolescents, and children**. *Food and chemical toxicology : an international journal published for the British Industrial Biological Research Association* 2017, **109**(Pt 1):585-648.

| **Reason for exclusion** | Wrong study design |
| --- | --- |

228. Winsloe C et al: **Early life factors in the pathogenesis of osteoporosis**. *Current osteoporosis reports* 2009, **7**(4):140-144.

| **Reason for exclusion** | Wrong study design |
| --- | --- |

229. Winzenberg T, Jones G: **Vitamin D and bone health in childhood and adolescence**. *Calcified tissue international* 2013, **92**(2):140-150.

| **Reason for exclusion** | Wrong study design |
| --- | --- |

230. Zheng J et al: **Maternal nutrition and the developmental origins of osteoporosis in offspring: Potential mechanisms and clinical implications**. *Experimental biology and medicine (Maywood, NJ)* 2018, **243**(10):836-842.

| **Reason for exclusion** | Wrong study design |
| --- | --- |
