# Supplementary material for: Nutrients, Diet, and Other Factors in Prenatal Life and Bone Health in Young Adults: A Systematic Review of Longitudinal Studies
Source: Nutrients. 2020 Sep 19;12(9):2866. doi: 10.3390/nu12092866 (PMC7551661; doi:10.3390/nu12092866)
Supplement: Supplementary file 1 [file nutrients-12-02866-s001.zip › Table S4.docx]

**Table S4:** Additional descriptions of the included studies in “Nutrients, Diet, and Other Factors in Prenatal Life and Bone Health in Young Adults: A Systematic Review of Longitudinal Studies”

| 1^st^ Author | BMI (kg/m^2^), offspring | Gender, Offspring  (% male) | Ethnicity, maternal | Maternal BMI, pre-pregnancy | Comparator | Method  Calibrated (+) / No information (NI) |
| --- | --- | --- | --- | --- | --- | --- |
| Hannam | -  (only height and weight are available) | 45.9% | - | Underweight (<18.5):  n = 138  Normal (18.5-24.9):  n = 2423  Overweight (25.0-30.9): n = 400  Obese (>30):  n = 127 | GH/PE vs. no HDP | DXA  (NI) |
| Jones | -  (only height and weight are available) | Never breastfeed: 61%  Ever breastfeed: 65% | - | - | ^1^ Smoking vs. no smoking | DXA  (+) |
| Martínez-Mesa | (Mean (s.e))  Males: 23.2 (0.10)  Females: 23.4 (0.11) | 49.2% | White: 76.6%(males) and 78.4%(females)  Non-white: 23.4%(males) and 21.6%(females) | -  (only height and weight are available) | ^2^ Number of cigarettes smoked per day | DXA  (+) |
| Miettola | (Mean±SD)  VLBW:  PE: 22.9 ± 3.7  No PE: 22.0 ± 3.7  Term:  PE: 23.7 ± 3.2  No PE: 23.2 ± 3.6 | 40.6% | - | (Mean±SD)  VLBW:  PE: 23.1±3.4  No PE: 22.1±3.8  Term:  PE 23.0±3.1  No PE 22.3±3.6) | PE vs. no HDP | DXA  (NI) |
| Rudäng | -  (only height and weight are available) | 100% | - | -  (only height and weight are available) | High maternal age (>36 years) vs. younger (<36 years) and age as a continuous variable | DXA, pQCT  (NI) |
| Yin | -  (only height and weight are available) | 70% | - | - | ^3^ Nutrient density | DXA  (+) |
| Zhu | (Mean±SD)  Males: 23.7 ± 3.7  Females: 23.6 ± 4.6 | 40.2% | Caucasian: 89.1%  Other: 10.9% | -  (only height and weight are available) | Vitamin D <50nmol/l vs. >50nmol/l and as a continuous variable (nmol/l). | DXA  (+) |

BMI, Body mass index; n, number; GH, Gestational Hypertension; PE, Preeclampsia; No HDP, no hypertensive disorders of pregnancy, VLBW, very low birth weight; Term, offspring born at term.

^1^ Maternal smoking in any trimester of pregnancy, measures by an administered postnatal questionnaire while mother and baby were in the hospital.

^2^ Numbers of cigarettes smoked per day reported by mothers in a questionnaire. Analyzed as a continuous variable.

^3^ i.e. protein, fat, carbohydrate, calcium, magnesium, phosphorus, fish, fruit, milk, meat, vegetable
